# Artificial intelligence-based endoscopic diagnosis of colorectal polyps using residual networks

**DOI:** 10.1371/journal.pone.0253585

**Published:** 2021-06-22

**Authors:** Yoriaki Komeda, Hisashi Handa, Ryoma Matsui, Shohei Hatori, Riku Yamamoto, Toshiharu Sakurai, Mamoru Takenaka, Satoru Hagiwara, Naoshi Nishida, Hiroshi Kashida, Tomohiro Watanabe, Masatoshi Kudo

**Affiliations:** 1 Department of Gastroenterology and Hepatology, Kindai University Faculty of Medicine, Osaka, Japan; 2 Faculty of Science and Engineering, Kindai University, Osaka, Japan; 3 Research Institute for Science and Technology, Kindai University, Osaka, Japan; 4 Cyber Informatics Research Institute, Kindai University, Osaka, Japan; University of Craiova, ROMANIA

## Abstract

Convolutional neural networks (CNNs) are widely used for artificial intelligence (AI)-based image classification. Residual network (ResNet) is a new technology that facilitates the accuracy of image classification by CNN-based AI. In this study, we developed a novel AI model combined with ResNet to diagnose colorectal polyps. In total, 127,610 images consisting of 62,510 images with adenomatous polyps, 30,443 with non-adenomatous hyperplastic polyps, and 34,657 with healthy colorectal normal mucosa were subjected to deep learning after annotation. Each validation process was performed using 12,761 stored images of colorectal polyps by a 10-fold cross validation. The efficacy of the ResNet system was evaluated by sensitivity, specificity, positive predictive value (PPV), negative predictive value (NPV), and diagnostic accuracy. The sensitivity, specificity, PPV, NPV, and diagnostic accuracy for adenomatous polyps at WLIs were 98.8%, 94.3%, 90.5%, 87.4%, and 92.8%, respectively. Similar results were obtained for adenomatous polyps at narrow-band imagings (NBIs) and chromoendoscopy images (CEIs) (NBIs vs. CEIs: sensitivity, 94.9% vs. 98.2%; specificity, 93.9% vs. 85.8%; PPV, 92.5% vs. 81.7%; NPV, 93.5% vs. 99.9%; and overall accuracy, 91.5% vs. 90.1%). The ResNet model is a powerful tool that can be used for AI-based accurate diagnosis of colorectal polyps.

## Introduction

Differentiating between a diagnosis of adenomatous and non-adenomatous hyperplastic polyps is important since the former type, which has the potential to develop into colorectal cancers, is an indication for endoscopic resection [1–3]. Recent advances in colonoscopic examination, including narrow-band imaging (NBI), have enabled endoscopists to diagnose adenomatous and non-adenomatous hyperplastic polyps without performing pathological examinations [4, 5]. However, it should be noted that even experienced endoscopists sometimes encounter difficulties in discriminating between adenomatous and non-adenomatous hyperplastic polyps [6]. Therefore, the establishment of an automatic diagnosis system for colorectal polyps is useful for endoscopists to perform endoscopic removal of adenomatous polyps and avoid unnecessary endoscopic resection for non-adenomatous hyperplastic polyps. Artificial intelligence (AI) is a valuable tool that can be used to accurately diagnose colorectal polyps. In fact, AI-based diagnosis for colorectal polyps has the potential to provide rapid and accurate differentiation between adenomatous and non-adenomatous hyperplastic polyps, as shown by recent studies [7–13].

Convolutional neural networks (CNNs) are widely used for AI-based image detection and classification [14]. The introduction of the CNN model into AI-based image diagnosis increases its sensitivity and specificity by promoting the deep learning process [14]. Completed CNN architectures comprise the following steps: the first convolution layer extracts features from input images; the second pooling layer reduces the number of image parameters, and the final fully connected layer-like neural network [14]. In completed CNN architectures, it is possible to add many convolution layers to achieve a high rate of accurate image classification. It has been generally accepted that deeper CNNs facilitate AI-mediated image classification accuracy [14]. However, recent reports provide evidence that CNNs with a greater depth do not always result in higher accuracy owing to the vanishing and exploding of image information gradients. A residual network (ResNet) is a new technology to overcome these defects in CNN-based AI. The ResNet achieves deeper learning than the conventional CNN system through the introduction of a shortcut connection, which prevents gradient loss and information degradation in multi-layer networks. ResNet is currently being recognized as a powerful tool for AI-based image classification [15]. This new technology has been applied in AI-based endoscopic diagnosis, as shown in recent publications [16, 17].

In this study, we developed a novel AI model combined with ResNet for the diagnosis of colorectal polyps. In the previous study, the overall accuracy of colorectal adenomatous polyps by inexperienced doctors was 75%, whereas that by experienced doctors was 89% [18]. Based on this report, we considered the overall accuracy of over 90% sufficient for the clinical application of AI-based colorectal polyp diagnosis. This novel ResNet-based AI model diagnosed colorectal adenomatous polyps with an accuracy of >90% in the validation *ex vivo* analysis. This is an innovative report that addresses the feasibility and utility of ResNet-based AI diagnosis of colorectal polyps.

## Materials and methods

### First-generation model of AlexNet system algorithm

We previously reported the diagnostic accuracy of AI-based colorectal polyps using AlexNet-based CNNs [19]. The AlexNet system is one of the conventional CNN algorithms [20]. AlexNet was established in 2012. A 7-layer network comprising a convolutional layer, pooling layer, and normalization layer was included.

The accuracy of a 10-cv validation in previous studies using AlexNet-based CNNs was 0.751 for the diagnosis of adenomatous and non-adenomatous hyperplastic colorectal polyps with a size of <1 cm. Such unsatisfactory results may be attributable to the limited number of images subjected to deep learning (a total of 1,800 images).

### Second-generation model of AlexNet system algorithm

**Fig 1:**
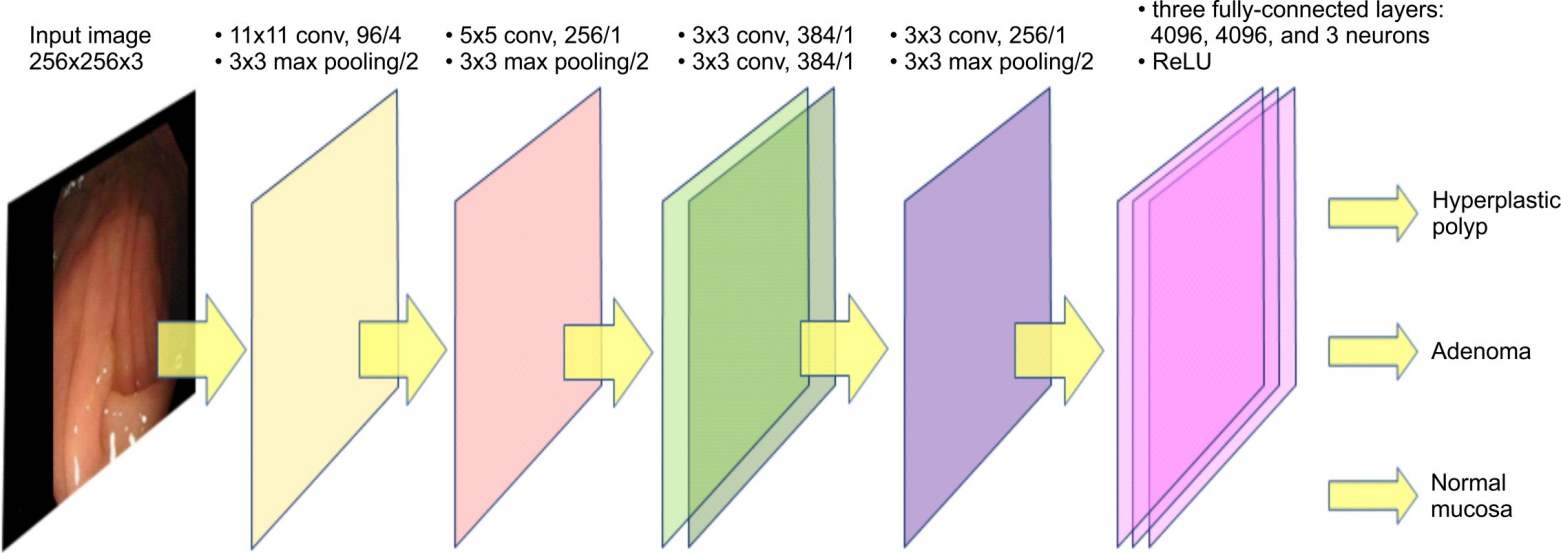
Architecture of AlexNet system (second-generation model).

To improve the accuracy of AI-based endoscopic diagnosis of colorectal polyps, we added healthy colorectal normal mucosal images to the first-generation model and increased the total number of learning images (Fig 1).

**Fig 2:**
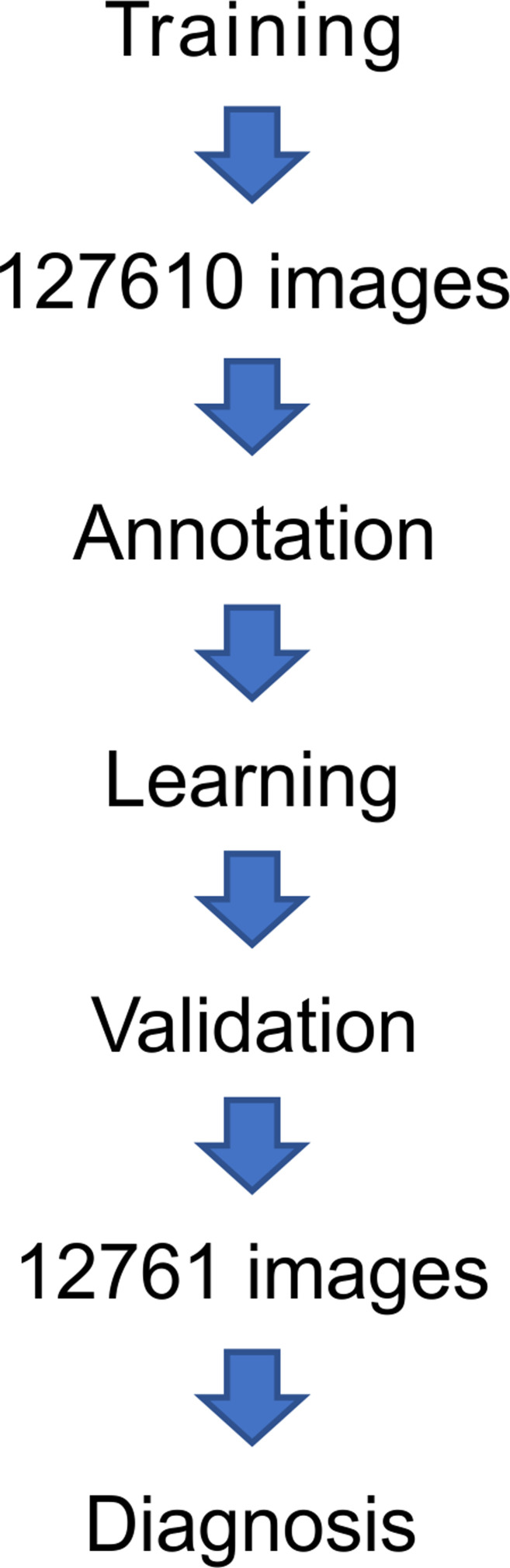
Flow chart of the study design.

These images were selected in a systematic order. There was no selection bias as these images were not manually selected. In the preparation of a set of training images, a total of 127,610 images, consisting of 62,510 images with adenomatous polyps, 30,443 images with non-adenomatous hyperplastic polyps, and 34,657 images with healthy colorectal normal mucosa obtained from 146 patients (146 polyps and surrounding healthy colorectal mucosa) were subjected to deep learning after annotation (Table 1). We performed a 10-fold cross validation (Fig 2). Each validation process was performed using 12,761 stored images of colorectal polyps (Fig 2). Training cases consisted of 29,810 white light images (WLIs), 37,500 NBI images, and 60,300 chromoendoscopy images (CEIs) for the second model of the AlexNet system. However, the overall accuracy of diagnosing colorectal polyps was less than 90% in WLIs, NBIs, or CEIs observed in the second-generation model of the AlexNet system.

**Table 1:** Characteristics of the polyps.

Training data set			
	Adenoma	Hyperplastic polyp	Total
Total no. of polyps	74	72	146
Polyp size (mm) (mean±SD)	5.2 (1.0)	4.8 (1.0)	5.0 (1.0)
Protruded shaped	54	47	101
Flat shaped	20	25	45

### ResNet system algorithm

To improve the accuracy further, we attempted to develop a novel AI-based system using ResNet, which is a new technology for AI-based image classification. Our major concern regarding AI-based image classification is that CNNs with a greater depth do not always result in higher accuracy, due to vanishing and exploding of image information gradients. We hypothesized that image information degradation associated with deeper layers might be involved in the overall accuracy with <90% in our second-generation model of the AlexNet system. To reduce image degradation, we tried to apply the ResNet system in the second-generation model. The "skip structure", a prominent feature of the ResNet system, allows the realization of a depth of 18 layers. In the skip structure, the input to a certain layer is bypassed, and subsequently, the signal is consequently directly input to the inner layer across the layers; this process prevents the disappearance and divergence of the gradient and achieves an ultra-multilayer network.

### Architecture of the ResNet system

**Fig 3:**
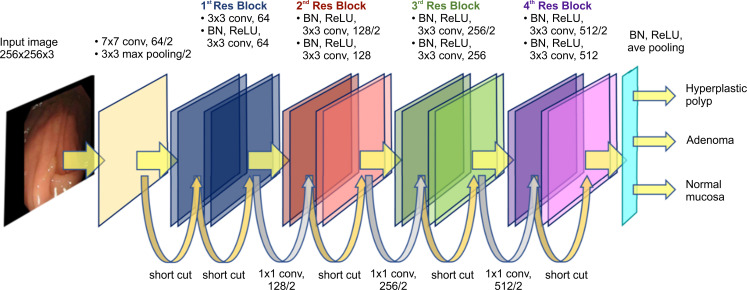
Architecture of ResNet system.

The sizes of the images for the ResNet-based CNN system were adjusted to 256×256 pixels. We first decided that the original image should be 1,920 x 1,080 pixels, and the size, after the image part of the endoscope was cut out, was adjusted to 256 × 256 pixels. We avoided characters, such as identification (ID) and patient names on the monitor. The smaller the input image size, the more efficient it is for the neural network to learn. However, if the image is too small, the performance will decrease; therefore, we ultimately chose 256×256 pixels to be the image size. The number of units in the input layer was equal to the size of the images, i.e., 256×256, and 64 convolution operators with 7×7 window size were applied to the input layer, followed by the pooling operator. After these operations, the number of units became 63×63×64. We incorporated four Res-blocks of the ResNet in our model to prevent the degradation of image information through their shortcut connection functions, in which each Res-block comprised two convolution layers and two rectified linear unit (ReLU) layers. The window size of the convolution operators in the convolution layers in the Res-Blocks was 3×3. The numbers of convolution operators in the four Res-Blocks were 64, 128, 256, and 512. After the incorporation of the four Res-Blocks, averaging pooling was applied. The fully connected layer finally yielded three outputs. Adam with a mini-batch size of 150 was employed. The learning rate was 0.001 and was divided by 10^1/2^. The models were trained with up to 20 iterations. Training with several images (127,610) from 146 patients finally led us to establish a novel ResNet system composed of a convolution and pooling layer and four Res-Blocks (Fig 3). To compare the diagnostic accuracy of our second AlexNet system and ResNet system, the same training and validation images are used as described in Fig 2 since we tried to compare the two models directly. This new AI system was validated using a 10-fold cross validation. In this 10-fold cross validation, adenoma, hyperplastic polyp, and normal mucosa were randomly divided into ten groups, and nine groups at three classes (WLI, NBI, and CEI) were used as training data. The remaining group was subjected to validation at three classes (WLI, NBI, and CEI). Image and poly count data together with standard deviation over the 10 folds are shown in Tables 1–4.

**Table 2:** The number of images with WLI, NBI, and CEI in data.

	Adenoma	Hyperplastic polyp	Healthy colorectal normal mucosa	Image count
WLI	9,238	6,910	13,662	29,810
NBI	19,068	14,069	4,363	37,500
CEI	34,204	9,464	16,632	60,300
Total	62,510	30,443	34,657	127,610

Training data set in 146 patients

Adenoma: 74 patients; Hyperplastic polyp: 72 patients

Healthy colorectal normal mucosa: surrounding mucosa of adenoma or hyperplastic polyp

**Table 3:** Diagnostic performance for adenomatous polyps in the AlexNet system of the second-generation model.

	WLI	NBI	CEI
**Sensitivity**	80.6%	93.4%	93.0%
**(99%CI)**	79.5–81.7	92.9–93.9	92.6–93.4
**Standard Deviation**	4.7	1.1	1.0
**Specificity**	78.5%	91.6%	84.7%
**(99%CI)**	77.2–79.8	91.0–92.2	83.7–85.7
**Standard Deviation**	3.6	1.6	2.7
**PPV**	79.1%	90.2%	90.3%
**(99%CI)**	78.0–80.2	89.7–90.7	89.9–90.7
**Standard Deviation**	4.4	1.6	1.1
**NPV**	74.5%	90.1%	90.5%
**(99%CI)**	73.2–75.8	89.4–90.8	89.7–91.3
**Standard Deviation**	2.9	1.1	2.8
**Overall Accuracy**	80.2%	89.0%	88.3%
**(99%CI)**	79.6–80.8	88.6–89.4	88.0–88.6
**Standard Deviation**	0.7	0.6	0.3

WLI: white light image, NBI: narrow-band image, CEI: chromoendoscopy image, PPV: positive predictive value, NPV: negative predictive value

**Table 4:** Diagnostic performance for the adenomatous polyps in the ResNet system.

	WLI	NBI	CEI
**Sensitivity**	98.8%	94.9%	98.2%
**(99%CI)**	98.5–99.1	95.5–95.3	98.0–98.4
**Standard Deviation**	2.9	0.7	3.0
**Specificity**	94.3%	93.9%	85.8%
**(99%CI)**	93.6–95.0	93.4–94.4	84.9–86.7
**Standard Deviation**	1.6	1.0	0.9
**PPV**	90.5%	92.5%	81.7%
**(99%CI)**	89.7–91.3	92.2–93.0	81.1–82.3
**Standard Deviation**	3.1	1.0	0.8
**NPV**	87.4%	93.5%	99.9%
**(99%CI)**	86.4–88.4	93.0–94.0	99.8–99.97
**Standard Deviation**	3.2	1.0	1.3
**Overall Accuracy**	92.8%	91.5%	90.1%
**(99%CI)**	92.4–93.1	91.1–91.9	89.8–90.4
**Standard Deviation**	2.8	0.5	1.7

WLI: white light image, NBI: narrow-band image, CEI: chromoendoscopy image, PPV: positive predictive value, NPV: negative predictive value

### Dataset section

Table 1 shows patients’ characteristics used in the training and final validation. Table 2 shows the numbers of images used for the training and final validation. These images comprised WLI, NBI, and CEI, as indicated. The same training and validation sets were used for both AlexNet and ResNet-based AI models. Diagnosis from the captured video was performed by real-time processing of the ResNet system, and the real-time diagnostic rate among adenomatous and non-adenomatous hyperplastic polyps and healthy colorectal normal mucosa was expressed on the monitor every 0.3 second (S1 Video). The durations of the videos were 30 seconds (shortest), 30 minutes and 26 seconds (longest), and 1 minute and 29 seconds (median).

### Polyp recognition

**Fig 4:**
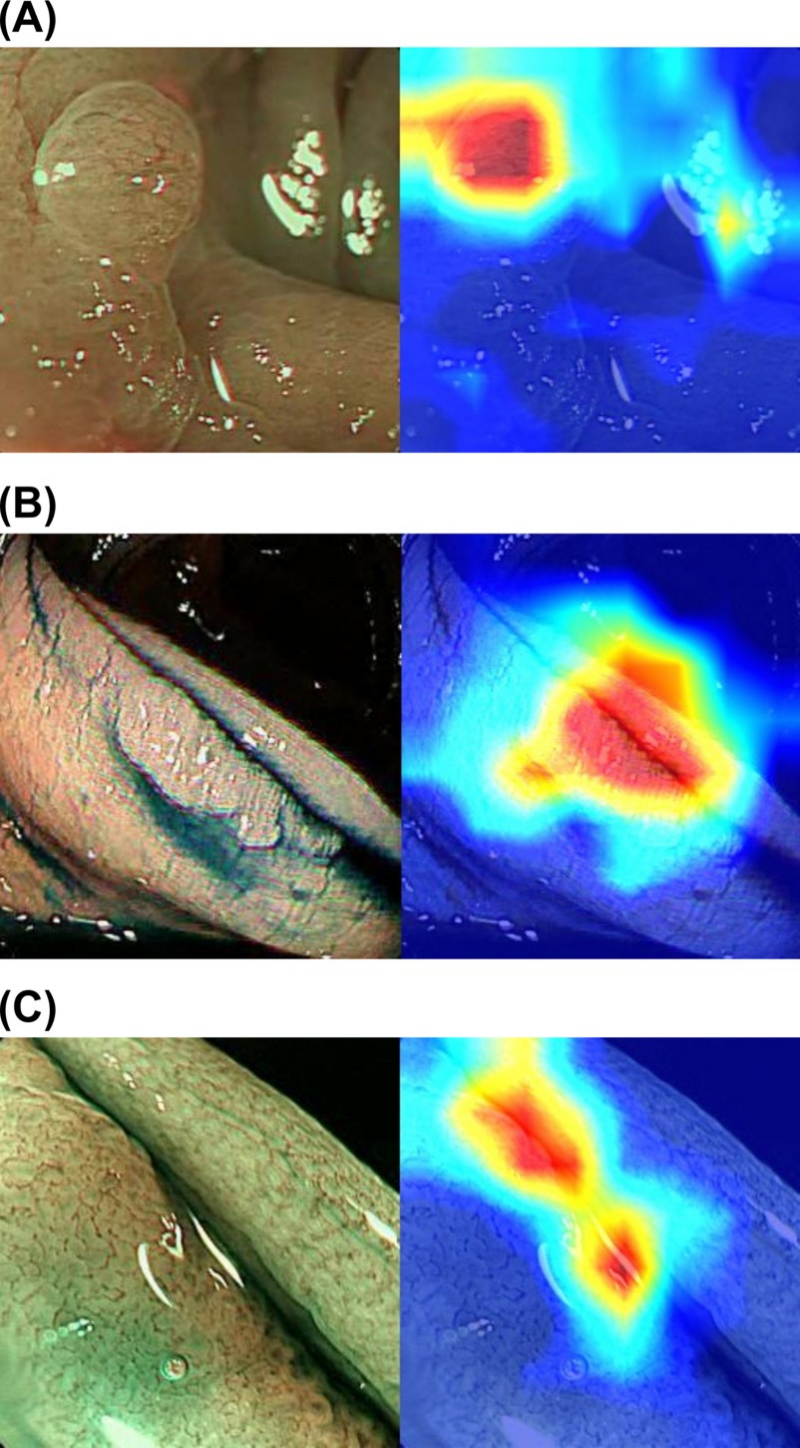
Polyp recognition. (A) A case of a non-adenomatous hyperplastic polyp. (B) A case of an adenomatous polyp. (C) A case of an adenomatous polyp.

The Gradient-weighted Class Activation Mapping (Grad-CAM) is used to retrieve the activated neurons from learned Neural Network models, where gradient-information is used for such retrievals [21]. Regarding Grad-CAM, the red to blue heat map shows which part of the image has more influence on the identification of the polyp **(**Fig 4A–4C). Representative heat map images of adenomatous polyps, non-adenomatous hyperplastic polyps were shown.

### Endoscopic evaluation

The diagnosis of colorectal polyps (adenomatous versus non-adenomatous hyperplastic) was based on the results of the pathological examinations. These images, including WLI, NBI, and CEI, were captured by a video endoscopy system (EVIS LUCERA ELITE system: Olympus Medical Systems, Co. Ltd, Tokyo, Japan) using colonoscopes (CF-H260AZI, CF-H260I, CF-260AI, and PCF-Q260AI: Olympus Medical Systems) during routine colonoscopic examination. These images were extracted from routine colonoscopies at Kindai University Hospital. Ethical permission for this study was granted by the review boards of Kindai University Hospital. Informed consent was not obtained because of the retrospective design of the study.

### Statistical analysis

Adenoma and hyperplastic polyps were finally diagnosed by pathological examinations. Based on the pathologically-diagnosed polyp-based analysis, we determined the sensitivity, specificity, and overall accuracy. Sensitivity, specificity, positive predictive value (PPV), negative predictive value (NPV), and overall accuracy were calculated according to the formula below and shown with 99% confidence interval (CI).

Sensitivity = Total number of images of second AlexNet or ResNet diagnosed as adenoma / Total number images of adenoma.

Specificity = Total number of images of second AlexNet or ResNet diagnosed as hyperplastic polyp / Total number images of hyperplastic polyp.

PPV = Total real adenoma images in total number images of second AlexNet or ResNet diagnosed as adenoma / Total number images of second AlexNet or ResNet diagnosed as adenoma.

NPV = Total real hyperplastic images in total number images of second AlexNet or ResNet diagnosed as hyperplastic polyp / Total number images of second AlexNet or ResNet diagnosed as hyperplastic.

Overall accuracy = Total real adenoma images in total number images of second AlexNet or ResNet diagnosed as adenoma polyp + Total real hyperplastic images in total number images of second AlexNet or ResNet diagnosed as hyperplastic polyp + Total real normal mucosa images in total number images of second AlexNet or ResNet diagnosed as normal mucosa / Total all images.

## Results

### The diagnostic rate of the second-generation model of AlexNet system algorithm

The efficacy of the second-generation model using the AlexNet system was evaluated through sensitivity, specificity, PPV, NPV, and overall accuracy analyses. In this study, the sensitivity, specificity, PPV, NPV, and overall accuracy for adenomatous polyps on WLIs were 80.6% (99%CI 79.5–81.7), 78.5% (99%CI 77.2–79.8), 79.1% (78.0–80.2), 74.5% (73.2–75.8), and 80.2% (79.6–80.8), respectively. Similar results were obtained for NBI and CEI (NBIs versus CEIs, sensitivity: 93.4% (99%CI 92.9–93.9) vs. 90.3% (92.6–93.4), specificity: 91.6% (99%CI 91.0–92.2) vs. 84.7% (99%CI 83.7–85.7), PPV: 90.2% (99%CI 89.7–90.7) vs. 90.3% (99%CI 89.9–90.7), NPV: 90.1% (99%CI 89.4–90.8) vs. 90.5% (99%CI 89.7–91.3), diagnostic accuracy: 89.0% (99%CI 88.6–89.4) vs. 88.3% (99%CI 88.0–88.6) (Table 3).

The diagnostic rate of the second-generation model displayed significant progress compared with that of the first-generation model [20]. However, the overall accuracy for colorectal polyps was <90% on WLIs, NBIs, and CEIs in the second-generation model of the AlexNet system and our previous first-generation model (75.1%).

### The diagnostic rate of the new ResNet system algorithm

The efficacy of the ResNet system was evaluated through the assessment of sensitivity, specificity, PPV, NPV, and overall accuracy analyses. In this study, the sensitivity, specificity, PPV, NPV, and overall accuracy for adenomatous polyps on WLIs were 98.8% (99%CI 98.5–99.1), 94.3% (99%CI 93.6–95.0), 90.5% (99%CI 89.7–91.3), 87.4% (86.4–88.4), and 93.5% (99%CI 92.4–93.1), respectively (Table 4).

Similar results were obtained for NBI and CEI (NBIs versus CEIs, sensitivity: 92.5% vs. 85.8%, specificity: 94.9% (99%CI 95.5–95.3) vs. 98.2% (99%CI 98.0–98.4), PPV: 92.5% (99%CI 92.2–93.0) vs. 81.7% (99%CI 81.1–82.3); NPV: 93.5% (99%CI 93.0–94.0) vs. 81.7% (99%CI 99.8–99.97), overall accuracy: 91.5% (99%CI 91.1–91.9) vs. 90.1% (99%CI 89.8–90.4) (Table 4). Thus, the accurate AI-based diagnosis of colorectal adenomatous polyps was achieved by this ResNet system.

The execution time of the ResNet model was 48.74 millisecond, whereas that of the AlexNet model was 41.59 millisecond. The diagnosis time is almost the same in the real world.

## Discussion

In this study, we tried to establish a novel AI-based endoscopic diagnosis system for colorectal polyps. Therefore, we used the ResNet system that enables the establishment of deeper CNNs without deterioration in accuracy. In a previous study, the overall accuracy of colorectal adenomatous polyps was 75% and 89% by inexperienced and experienced doctors, respectively. Based on this report, we consider an overall accuracy of over 90% sufficient for the clinical application of AI-based colorectal polyp diagnosis [18]. This ResNet-based AI diagnosis for colorectal polyps achieved a high overall accuracy of >90% in the discrimination between adenomatous and non-adenomatous hyperplastic polyps. This is an innovative study in which the ResNet-based CNN is applied to AI-based diagnosis of colorectal polyps, as in other recent publications [16, 17].

We previously reported the diagnostic accuracy of AI-based colorectal polyps using AlexNet-based CNNs [20]. The AlexNet system is one of the conventional CNN algorithms [21]. The overall accuracy of our previous studies was 0.751 for the diagnosis of adenomatous and non-adenomatous hyperplastic polyps in the first-generation model of the AlexNet system. Such unsatisfactory results can be partially explained by the limited number of images subjected to deep learning (1,800 images). To improve the accuracy of AI-based endoscopic diagnosis of colorectal polyps, we added healthy colorectal normal mucosal images to the first-generation model and increased the total number of learning images. However, the overall accuracy for colorectal polyps was less than 90% on WLIs, NBIs, and CEIs in the second-generation model of the AlexNet system. Thus, the AlexNet system is not independently sufficient to achieve a satisfactory overall accuracy for colorectal polyps using AI.

To further facilitate the diagnostic accuracy, we introduced the new ResNet system in this study. The ResNet system achieves deeper learning than the conventional CNN system through the introduction of a shortcut connection, which prevents gradient loss and information degradation in multi-layer networks. An overall accuracy of >90% was successfully achieved by the ResNet system. We speculate that the improvement in the overall accuracy might be partially explained by the prevention of image degradation by the ResNet system. Our new ResNet model was able to efficiently differentiate adenomatous polyps from non-adenomatous hyperplastic polyps as demonstrated in our findings that the sensitivity, specificity, PPV, NPV, and overall accuracy exceeded 90% on WLIs, NBIs, and CEIs compared with recent publications [16, 17].

Regarding the slightly lower sensitivity and NPV on CEIs, we speculate that the artifact of the indigo-carmine fluid made it difficult to recognize the polyps. On the contrary, both AlexNet-based and ResNet-based AI models achieved comparable diagnostic performance. In addition, WLI mode was found to have better sensitivity and specificity than NBI/CEI mode in the ResNet in our final analysis. For the AI diagnosis based on the ResNet system, there is a possibility that WLI might be sufficient, and NBI might be unnecessary. The reason such a high performance was achieved using WLI, but not with NBI, remains unknown. Therefore, these data need to be verified in multicenter studies prospectively.

Many researchers have developed their own AI models for the endoscopic diagnosis of colorectal polyps [7–13, 22]. The diagnostic utility of our ResNet model did not appear to be superior or inferior to these previous models since the sensitivity, specificity, PPV, NPV, and overall accuracy in all of these studies, including ours, are comparable. Recently, three studies reported the overall accuracy of AI-based colorectal polyps using the CNNs [9, 13, 22]. All these models used deep CNNs without the ResNet system. Regarding the sensitivity, specificity, NPV, and PPV, no significant differences were observed in the diagnostic utility in these studies, including ours [9, 13, 22]. Thus, the final data of these parameters for the evaluation of the overall accuracy were comparable to whether deep CNN-based AI models have been combined with the ResNet system or not for the detection of adenomatous colorectal polyps. In a direct comparison of the ResNet system with the AlexNet system, we confirmed that the former technology was superior to the latter technology in terms of all the parameters examined. However, it is too early to determine the superiority and inferiority of these models with or without the ResNet system since the diagnostic utility was assessed by *ex vivo* analysis in all these studies, including ours. Therefore, further research on these models in a clinical trial (using a live patient) is necessary to determine whether our ResNet-based AI is a powerful diagnostic tool for colorectal polyp diagnosis.

Misawa et al. and Mori et al., [7, 8, 10, 11] were the pioneers that tried to investigate the computer-aided diagnosis (CAD) of colorectal polyps. Their model utilized endocytoscopy by which highly magnified nuclear images were obtained in combination with chromoendoscopy. They established the CAD system employing endocytoscopy through the application of highly magnified nuclear images in the learning process. Although the CAD system employing endocytoscopy has already been tested in the real-time diagnosis of colorectal polyps with a very high pathologic prediction rate (98.1%), a very limited number of hospitals presently utilize endocytoscopy. On the contrary, our ResNet model utilizes routine colonoscopies rather than endocytoscopy. Given the fact that our model achieves a high overall accuracy in the *ex vivo* analysis using colonoscopes for routine examinations, the ResNet model can be a powerful tool for AI-based diagnosis of colorectal polyps. If the diagnostic ability is confirmed in future studies, the ResNet system is expected to reduce medical costs. This is because the application of this technology is certain to reduce the rates of unnecessary polypectomies.

This study has several limitations. First, it was a retrospective study in a single university hospital. Second, this study was performed *ex vivo*. In the validation study, the ResNet model diagnosed the endoscopic images extracted from the video captured from routine colonoscopy images.

In conclusion, the ResNet model is a powerful tool for AI-based accurate diagnosis of colorectal polyps. This newly developed model can be used in routine colonoscopic examinations. However, it should be noted that future multicenter studies addressing the overall accuracy for colorectal polyps prospectively *in vivo* are warranted to confirm the efficacy of this novel technology.

## Supporting information

S1 Video Diagnosis from the captured video is performed by real-time processing of ResNet, and the real-time diagnostic rate for the hyperplastic polyps, adenomas, and healthy colorectal normal mucosa is expressed on the monitor every 0.3 seconds. (MP4)
